# Patient-Level Predictors of Psychiatric Readmission in Substance Use Disorders

**DOI:** 10.3389/fpsyt.2019.00828

**Published:** 2019-11-26

**Authors:** Volker Böckmann, Barbara Lay, Erich Seifritz, Wolfram Kawohl, Patrik Roser, Benedikt Habermeyer

**Affiliations:** ^1^Department of Addictive Disorders, Psychiatric Services Aargau, Brugg, Switzerland; ^2^Department of Psychiatry and Psychotherapy, Psychiatric Services Aargau, Brugg, Switzerland; ^3^Department of Psychiatry, Psychotherapy and Psychosomatics, Psychiatric Hospital, University of Zurich, Zurich, Switzerland

**Keywords:** readmission, substance use disorders, number of admissions, HoNOS, patient-level predictors

## Abstract

Repeated psychiatric readmissions are a particular challenge in the treatment of substance use disorders and are associated with substantial burden for patients and their associates and for healthcare providers. Factors affecting readmission rates are heterogeneous and need to be identified to better allocate resources. Within the Swiss healthcare system, such data on substance use disorder patients are largely missing. Understanding these factors might bear important implications for future healthcare planning. Thus here, we examine risk factors of inpatient readmission. We retrospectively analyzed all admissions to the hospital's department of addictive disorders in the year 2016. Patients included in the study were followed over a period of 1 year after discharge regarding readmissions to the clinic. Besides the demographic, social, and economic data, we extracted data concerning patient history, admission, and discharge as well as clinical data regarding type and number of substances abused and comorbid diagnoses. In order to describe severity of cases, we furthermore included the scores of the Health of the Nation Outcome Scale (HoNOS) at admission and at discharge as documented in the medical database. Of the 554 patients included in the study, 228 (41.2%) were readmitted within 12 months. Previous admissions, concomitant use of different substances, presence of psychosis or mania, and a higher severity score at discharge increased the likelihood of readmission. The odds for readmission were furthermore higher in patients not being married, living alone, and being unemployed. When all (bivariate) statistically significant factors are included into a logistic regression model, the previous number of admissions and the HoNOS clinical score at discharge significantly contributed to this model. Our findings stress that patients with higher symptom load at discharge are prone to be readmitted within 12 months. The same applies for patients with previous admissions. These findings suggest that the development of specific interventions to prevent premature discharge before satisfactory symptom remission, in particular in those patients with previous admissions in their patient history, might help to prevent readmissions.

## Introduction

Substance use disorders (SUDs) are conditions in which the use of legal or illicit substances leads to significant impairment or distress (1). According to the World Health Organization (2), SUDs are responsible for 39 deaths per 100,000 population and for 5% of the global burden of disease, as measured in disability adjusted life years (3). The impact of SUD also poses a challenge for the public healthcare systems. The mere annual costs in the US for the treatment of SUDs were estimated 100 billion dollars in 2003 (4), and a large proportion of these costs is related to hospital admissions for detoxification. A particular problem in psychiatric disorders in general and specifically in the treatment of SUDs are repeated readmissions and the so-called revolving door phenomenon (5). With regard to alcohol dependence, readmission rates between 30% within 6 months (6) and 51% within 1 year (7) are reported in the literature. Frequent readmissions are not only burdensome but they also contribute to stigmatization (8) and the overall treatment costs of SUDs. Unplanned readmissions are often more costly than planned admissions (9), a reason why numerous studies assessed the probability of readmission for specific groups of patients or diseases (10). Understanding factors that predict readmission would allow to better address the specific needs of the population at risk and to prevent costly inpatient treatment. In addition, readmission rates might serve as an indicator of the quality of care and could therefore be of particular relevance for healthcare policy makers (11). Finally, previous readmission rates in SUD are often considered to be a significant predictor of further relapse after inpatient treatment.

From a recent review on pre-discharge factors of readmission in general psychiatric patients, we know that the most consistent factor predicting readmission appears to be a previous hospitalization (11). With regard to SUDs, further factors have been reported in various studies. In a study from the US on elderly Medicare inpatients with SUDs (12), female patients and patients of Afro-American origin were more likely to be readmitted over a 4-year interval. These patients also yielded more previous admissions, comorbidities, and accidents involving poisoning, adverse drug reactions, or falls. A study from the Netherlands in this age group found that spending leisure time alone was associated with higher readmission rates (7).

Moreover, social factors such as being homeless or on health coverage through fee-for-service Medicaid in the US have been shown to be related to increased readmission rates (13). Other risk factors for readmission were alcohol as the primary drug of choice, residential instability, multiple drug use, single marital status, unemployment, being older than 37 years, and treatment dropout (14). Some of these factors were confirmed earlier in a small Swiss sample that identified living alone, single marital status, and pretreatment frequency of alcohol intake as predicting factors for readmission (15).

However, the abovementioned findings are inconsistent. One reason for this inconsistency might be that some statistical models did not control for previous admissions, i.e., the variable most consistently found to be associated with readmission in psychiatric patients. On the other hand, the inconsistent findings might be explained by differences in the healthcare systems of the countries in which the studies were conducted. For example, Switzerland only has a very small proportion of patients with SUDs that are homeless or have no access to inpatient treatment compared to the US or other European countries.

It is therefore important to identify those patients with SUDs at high risk of readmission to inpatient treatment and to better understand the factors that contribute to this risk.

Factors that may affect readmission rates are manifold and can be divided into different categories. Specifically, factors at the patient level (e.g., sex, age, and disease severity) can be differentiated from factors at the program level (e.g., availability of treatment, type, and setting of treatment). In this study, we primarily focused on predicting factors at the patient level. As far as we are aware of, our study is the first to address this question in more detail on the basis of a larger sample of consecutively referred patients to a specialized SUD service followed over the period of 12 months.

## Methods

### Setting and Sample

The Psychiatric Services Aargau (PDAG) are an academic teaching hospital of the University of Zurich, Switzerland, and provides a capacity of 331 inpatient treatment places for adults with acute or chronic psychiatric disorders of all kinds. The hospital provides inpatient treatment for approximately 700,000 inhabitants of the Canton of Aargau, Switzerland. The Department of Addictive Disorders of the PDAG is the only provider for acute psychiatric inpatient treatment for patients with SUDs in the canton. The department consists of three separate wards, of which one provides 11 places with a closed treatment setting. On this ward, another 11 patients can be treated in an open setting, and the other two wards provide additional 42 treatment places in an open setting.

We performed a retrospective chart review using the electronical medical records from all patients that were discharged from the Department of Addictive Disorders within the period from January 2016 to December 2016. All patients with a diagnosis of the ICD category F10–F19 of mental and behavioral disorders due to psychoactive substance use were included into *post hoc* analysis of data. For every patient included in the study, a follow-up period of 1 year after the day of discharge was defined. We analyzed features of the index episode and the next readmission within this 1-year follow-up period.

The study was approved by the responsible regional ethics committee (BASEC 2017-01533).

### Data Collection

We retrieved information on the following factors from the database of the PDAG:

Demographic (age and gender), social [marital status: married vs. not married, separated, or widowed; living situation: together with others vs. alone or in an institution; education: compulsory schooling (corresponds to 11 years of schooling), unknown vs. high school, apprenticeship (15 years; apprenticeship corresponds to a specific dual education option with company-based training and part-time vocational schooling), or college/university (more than 15 years)], and economic (employment: employed vs. unemployed or on disability pension). We also extracted data concerning the patients' history (number of admissions), information on admission and discharge (length of stay, involuntary admission, and type of discharge: mutual consent vs. treatment provider's or patient's initiative). We furthermore included clinical data regarding substance of abuse, number of SUDs as well as additional psychiatric diagnoses. The Health of the Nation Outcome Scale (HoNOS) (16) at admission and at discharge retrieved from the medical database of the PDAG were used as indicators of clinical severity. The HoNOS is an established rating instrument for the assessment of severity in patients with mental disorders and consists of 12 items measuring four broader categories: behavior, impairment, symptoms, and social functioning. Each item can be rated from 0 to 4, resulting in total values between 0 and 48. According to the thresholds suggested by Parabiaghi et al. (17), total HoNOS scores over 13 are considered to indicate a severe stage of illness while lower scores indicate a rather moderate stage of illness.

### Data Analysis

To analyze the data, all patients with SUDs were classified into two groups: patients with and without readmission within 1 year after discharge. For scaled variables (like HoNOS or length of stay), the means and standard deviations (SD) were calculated. For categorical variables (like marital status or employment), the numbers (*n*) and percentages (%) were reported for the two groups. In order to identify potential predictors, we calculated logistic regression models and the Wald test was used to test the coefficients. We first calculated odds ratios for each of the abovementioned variables separately with readmission (no readmission vs. readmission within 1 year) as the dependent variable. All factors/variables that showed a statistically significant effect were then included together in a logistic regression model. Significance level was fixed at *p* < 0.05.

All analyses were performed with IBM SPSS Statistics for Windows, version 23.0 (IBM Corp., Armonk, NY).

## Results

Between January and December 2016, 566 patients were discharged from the Department of Addictive Disorders. Of these, 12 patients had to be excluded because they had no SUD diagnosis. The characteristics of the remaining 554 patients are reported separately for readmitted (*n* = 228) and not readmitted (*n* = 326) patients in **Table 1**. The total readmission rate was 41.2% within the first 12 months after discharge.

**Table 1:** Sample characteristics of all patients with substance use disorders not readmitted and readmitted within 1 year.

	Overall	Not readmitted	Readmitted	Wald (*df*)	*p* value	OR	CI (95%)
	554 (100%)	326 (58.8%)	228 (41.2%)				
**Demographic, social, and economic**							
Age, mean (SD)	40.5 (12.4)	41.0 (12.5)	39.7 (12.1)	1.505 (1)	0.220	0.991	0.98–1.01
Sex, *n* (%)							
Female*	166 (30%)	100 (60.2%)	66 (39.8%)				
Male	388 (70%)	226 (58.2%)	162 (41.8%)	0.191 (1)	0.662	1.086	0.75–1.57
Marital status, *n* (%)							
Married*	92 (16.6%)	65 (70.7%)	27 (29.3%)				
Not married, separated, widowed	462 (83.4%)	261 (56.5%)	201 (43.5%)	6.224 (1)	0.013	1.854	1.14–3.01
Living situation, *n* (%)				9.389 (2)	0.009		
Together, home*	238 (43.0%)	152 (63.94%)	86 (36.1%)				
Alone, home	227 (41.0%)	134 (59.0%)	93 (41.0%)	1.146 (1)	0.284	1.227	0.84–1.78
Institution	89 (16.1%)	40 (44.9%)	49 (55.1%)	9.380 (1)	0.002	2.165	1.32–3.55
Education, *n* (%)				3.126 (2)	0.201		
Compulsory schooling, unknown*	220 (39.7%)	123 (55.9%)	97 (44.1%)				
High school, apprenticeship	268 (48.4%)	158 (59.0%)	110 (41.0%)	0.459 (1)	0.498	0.883	0.62–1.27
College, university	66 (11.9%)	45 (68.2%)	21 (31.8%)	3.118 (1)	0.077	0.592	0.33–1.06
Employment, *n* (%)				10.440 (2)	0.005		
Employed*	142 (25.6%)	97 (68.3%)	45 (31.7%)				
Unemployed	283 (51.1%)	166 (58.7%)	117 (41.3%)	3.714 (1)	0.054	1.519	0.99–2.32
Disability pension	129 (23.3%)	63 (48.8%)	66 (51.2%)	10.440 (1)	0.001	2.258	1.38–3.70
**Patients' history**							
Number (%) of patients with				51.470 (3)	<0.001		
1 admission*	272 (49.1%)	198 (72.8%)	74 (27.2%)				
2 admissions	85 (15.3%)	48 (56.5%)	37 (43.5%)	7.890 (1)	0.005	2.062	1.25–3.42
3 admissions	56 (10.1%)	30 (53.6%)	26 (46.4%)	7.829 (1)	0.005	2.319	1.29–4.18
≥4 admissions	141 (25.5%)	50 (35.5%)	91 (64.5%)	50.572 (1)	<0.001	4.870	3.15–7.53
**Admission and discharge**							
Length of stay, mean (SD)	29.4 (21.9)	28.4 (20.6)	31.0 (23.6)	1.827 (1)	0.176	1.005	1.00–1.01
Involuntary admission, *n* (%)	113 (20.4%)	60 (53.1%)	53 (46.9%)	1.929 (1)	0.165	1.343	0.89–2.04
Type of discharge, *n* (%)				5.680 (2)	0.580		
Mutual consent*	384 (69.3%)	231 (60.2%)	153 (39.8%)				
On treatment provider's initiative	53 (9.6%)	23 (43.4%)	30 (56.6%)	5.238 (1)	0.022	1.969	1.10–3.52
On patient's initiative	117 (21.1%)	72 (61.5.0%)	45 (38.5%)	0.072 (1)	0.789	0.944	0.62–1.44
**Clinical**							
Substance, *n* (%)							
Alcohol	365 (65.9%)	207 (56.7%)	158 (43.3%)	2.009 (1)	0.157	1.298	0.90–1.86
Opioids	134 (24.2%)	80 (59.7%)	54 (40.3%)	0.054 (1)	0.817	0.954	0.64–1.41
Cannabis	101 (18.2%)	56 (55.4%)	45 (44.6%)	0.589 (1)	0.443	1.186	0.77–1.83
Benzodiazepines	83 (15.0%)	50 (60.2%)	33 (39.8%)	0.079 (1)	0.779	0.934	0.58–1.50
Cocaine/amphetamines	95 (17.1)	50 (52.6%)	45 (47.4%)	1.828 (1)	0.177	1.357	0.87–2.12
Number of substance use disorders, mean (SD)	1.5 (0.9)	1.4 (0.9)	1.6 (1)	4.793 (1)	0.029	1.222	1.02–1.46
Patients with dual diagnosis, *n* (%)	402 (72.6%)	233 (58.0%)	169 (42.0%)	0.473 (1)	0.492	1.143	0.78–1.68
Additional diagnoses, *n* (%)							
Psychosis/mania	49 (8.8%)	22 (44.9%)	27 (55.1%)	4.215 (1)	0.040	1.856	1.03–3.35
Depression	183 (33.0%)	107 (58.5%)	76 (41.5%)	0.016 (1)	0.900	1.023	0.71–1.47
Anxiety/adjustment/PTSD	91 (16.4%)	58 (63.7%)	33 (36.3%)	1.072 (1)	0.300	0.782	0.49–1.25
Personality disorder	113 (20.4%)	59 (52.2%)	54 (47.8%)	2.566 (1)	0.109	1.404	0.93–2.13
ADHS	48 (8.7%)	27 (56.3%)	21 (43.7%)	0.146 (1)	0.702	1.123	0.62–2.04
Number of additional diagnoses, mean (SD)	0.9 (0.7)	0.9 (0.7)	1 (0.7)	2.527 (1)	0.112	1.223	0.95–1.57
Additional diagnoses, *n* (%)							
≤1 additional diagnosis*	469 (84.7%)	283 (60.3%)	186 (39.7%)				
> 1 additional diagnosis	85 (15.3%)	43 (50.6%)	42 (49.4%)	2.804 (1)	0.095	1.486	0.94–2.36
HoNOS, mean (SD)							
At admission (index episode)	14.5 (5.6)	14.1 (5.5)	15.1 (5.7)	3.449 (1)	0.063	1.031	1.00–1.06
At discharge (index episode)	9.2 (5.4)	8.5 (5.3)	10.3 (5.4)	11.890 (1)	0.001	1.064	1.03–1.10

* reference category; df, degrees of freedom; OR, odds ratio; CI, confidence interval.

### Demographic, Social, and Economic Factors

Marital status showed to have an influence on readmission. Patients not married, separated, or widowed showed a higher readmission rate than patients who were married (OR = 1.854). Similarly, patients living alone and, even more so, patients living in institutions were more likely to be readmitted than patients living together with their family ("living alone" OR = 1.227, "living in institution" OR = 2.165, ref "together, at home"). Moreover, if patients were unemployed or on disability pension, the chance for readmission was elevated as compared to patients on employment ("unemployed" OR = 1.519, "disability pension" OR = 2.258, ref "employment"). Age, gender, and education level did not show significant effects on the risk of readmission.

### Patients' History

The likelihood of readmission increased with the number of previous admissions ("2 admissions" OR = 2.062, "3 admissions" OR = 2.319, "≥4 admissions" OR = 4.870, ref "1 admission").

### Admission and Discharge

The length of stay did not show a significant effect. Moreover, involuntary admission in the index episode and type of discharge in the index episode did not significantly increase the risk of readmission.

### Clinical Data

While the different substances used by the patients showed no effect on the readmission risk, the total number of SUDs was associated with a higher risk of readmission (OR = 1.222). A concomitant diagnosis of schizophrenic psychosis or bipolar mania increased the likelihood of readmission (OR = 1.856). And also with higher HoNOS scores at discharge the risk of readmission increased (OR = 1.064). HoNOS at admission did not show a statistical significant effect. When all statistically significant predictors are entered together in a logistic regression model, the number of "admissions" still revealed to be the factor most strongly associated with the risk of readmission (**Table 2**). The risk to be readmitted increased by a factor of 2 or more after the second admission (OR = 2.057), and patients with a history of four or more admissions had an even five times higher risk of readmission within the following 12 months (OR = 5.425). After controlling for the effect of the number of previous admissions, the HoNOS score further contributed to the model: Regarding the HoNOS score at discharge (index episode), findings suggest that the likelihood of readmission increased by 5.7% with each further point on the HoNOS scale (OR = 1.057). All other variables which showed significant effects in the bivariate analysis did not further contribute to this regression model (Nagelkerkes *R*^2^ = 0.199; Hosmer–Lemshow test: *X*^2^(8) = 8.255, *p* = 0.412).

**Table 2:** Risk factors of psychiatric readmission within 1 year.

Predictors	*B*	SE	Wald (*df*)	*p* value	Adjusted OR	CI (95%)
HoNOS at discharge	0.055	0.020	7.584 (1)	0.006	1.057	1.016–1.100
Admissions			39.629 (3)	0.001		
1 admission*						
2 admissions	0.721	0.300	5.774 (1)	0.016	2.057	1.142–3.705
3 admissions	0.836	0.336	6.181 (1)	0.013	2.307	1.194–4.460
≥4 admissions	1.691	0.270	39.342 (1)	0.001	5.425	3.198–9.202
Number of substance use disorders	0.038	0.116	0.107 (1)	0.744	1.039	0.828–1.303
Additional psychosis/mania	0.095	0.372	0.065 (1)	0.799	1.099	0.531–2.277
Marital status	0.205	0.305	0.453 (1)	0.501	1.228	0.675–2.232
Living situation			3.011 (2)	0.222		
Together, home*						
Alone, home	0.110	0.234	0.22 (1)	0.639	1.116	0.705–1.766
Institution	0.547	0.318	2.953 (1)	0.086	1.727	0.926–3.222
Employment			0.239 (2)	0.887		
Employed*						
Unemployed	0.044	0.281	0.024 (1)	0.876	1.045	0.602–1.813
Disability pension	0.149	0.329	0.205 (1)	0.650	1.161	0.609–2.214

* reference category. Nagelkerkes R^2^ = 0.199.

## Discussion

Our study aimed to identify potential social and clinical predictors of readmission in patients with SUDs. We analyzed readmission rates of patients consecutively referred to a large SUD department, which is responsible for the entire Canton of Aargau. Patients were followed over a period of 1 year.

In our study sample, 41.2% of the patients were readmitted to inpatient treatment. Compared to a recent Dutch study that applied the same observation period and found a readmission rate of 50.8% (7), we found less readmissions in our sample. Similar results have been obtained by another survey of a state Medicaid program in the US that reported a readmission rate of up to 47% in patients with SUDs within 1 year after detoxification (18). Lower readmission rates are often related to shorter observation periods. For example, Slater and Linn (6) reported a readmission rate of 30% for a male population treated for alcohol use disorder within the following 6 months, and (19) demonstrated readmission rates of 18% for alcohol-related disorders and 15% for drug-related disorders, respectively, within 30 days after discharge.

Interestingly, age and gender had no impact on the readmission risk in our study. This finding does not corroborate previous studies that consistently reported higher readmission rates in younger patients with SUDs (20). However, Moos et al. showed in another study (21) that this effect might also be influenced by program characteristics. According to the latter study, intensive and direct treatment appeared to be more effective in younger subjects, whereas more supportive settings in a highly organized program with immediate aftercare was more helpful for older subjects. In our study, the mean age of the patients was 40 years. Most studies that addressed the association between patients' ages and outcomes defined old age starting with 55 years and young age ending with 34 years (20, 21). Accordingly, our sample represents a middle-aged group. It can therefore be speculated that age-related differences are less common in this age group.

With regard to the marital status, a recent review pointed out that being married generally appears to be a protective factor against readmission of patients suffering from psychiatric diseases (11). According to the literature, this observation also applies to readmission in SUD (20–22). Our data confirm this notion that unmarried, separated, or widowed patients are more likely to be readmitted. Studies on psychotic patients showed that being single increases the risk of readmission, while others found that a divorce increases the likelihood of readmission (23). There is a large evidence that SUDs are generally associated with dysfunctional attachment (24). Relational difficulties in SUD are underscored by the fact that divorces are seven times more likely in patients with alcohol addiction than in others (25). While the direction of the association between interpersonal difficulties and SUD is not fully understood, some results indicate that dysfunctional attachment precedes SUD (24). However, not only interpersonal factors but also higher self-efficacy and less reliance on avoidance coping have also been demonstrated to prevent readmissions (20) and might contribute to successful relations. It can be speculated that patients still being in a marriage have (or have preserved) better interpersonal or coping skills and that these skills also contribute to a lower rate of readmission, while in single or separated patients interpersonal or coping problems are more pronounced.

This latter aspect is in line with our observation that the living situation, as defined in terms of place and household composition, appears to be significantly associated with the risk of readmission. In this context, patients that lived together with others had less readmissions compared to patients living alone or in institutions. From the literature, we know that homeless people with SUDs are prone to readmission to inpatient treatment (26). In Switzerland, however, the rate of homelessness is generally very low (approximately 300–500 per 8 million inhabitants) compared to other countries. In our sample, only 27 (4.9%) patients reported to be homeless. Because of this small number, we pooled this group with the subjects living alone. With regard to subjects living in institutions, we have some evidence from Swiss data that patients living in community housing facilities have even more problems concerning SUD, physical illness, and psychopathological symptoms compared to patients on an acute psychiatric ward (27). This finding might explain why living in an institution is associated with a higher likelihood of being readmitted to inpatient treatment.

In our sample, education was not associated with the risk of readmission. This finding is somewhat surprising as the National Survey on Drug Use and Health from the US Substance Abuse and Mental Health Services Administration (28) showed that individuals that graduated from high school or college/university showed lower rates of SUD than those that did not complete high school. Several studies demonstrated furthermore that patients with a higher educational level showed smaller readmission rates (e.g., 29). The missing effect of education on the readmission rate in our study might have to do both with the age range of our sample and with the type of substances of use. SUDs develop often gradually over many years. This process depends on various factors like time of first use, type of substance, or presence of other preexisting mental disorders (30). According to this study, this process is faster for patients using opioids and stimulants. In our sample, alcohol is by far the most frequently used substance. Due to the somewhat slower development of a manifested SUD in alcohol-related disorders, the years of school education might have been reprieved from the direct negative effects of SUD. In younger patients, family involvement, community consultation, and development of social and work skills were associated with lower readmission rates (21). Hence, it can be further speculated that the disruptive effects of SUD might have also been attenuated by the elaborated educational and social system of Switzerland, which provides a high social security and social support (e.g., every child has to attend school at least for 11 years and parents do not have to pay tuitions).

Previous studies reported that full-time employment may have strong protective effects against readmission to psychiatric inpatient treatment (31). Consistently, we could demonstrate that unemployed patients with SUDs or patients with SUDs receiving a disability pension were more likely to be readmitted compared to patients being in employment. According to Magura (32) and Platt (33), employment appears to be of high predictive value for a favorable outcome of SUD treatment. Behind a successful recovery of SUD, employment appears to be the second most important priority for the patients (34), and a high motivation to preserve an existing employment might contribute to our findings.

A patient's history with previous admissions appears to be the most important predictor of readmission. The present data suggest a five times higher risk of readmission to inpatient treatment in patients with more than three admissions in their patient history compared to those with only one admission. This finding is in line with a recent systematic review that could demonstrate that admission history was significantly associated with readmission in 32 out of 37 studies (11). Twenty of these studies were able to confirm this association by multivariate analyses indicating a rather robust finding. More admissions in the patient's history are often related to a longer duration of illness and previous use of health services, which are also risk factors for readmission (35).

Unexpectedly, our study did not reveal any influence of neither involuntary admission nor type of discharge on the risk of readmission. In contrast, a study from Israel on involuntary admission to SUD treatment suggested that coercion might at least delay (36) or even prevent readmissions (37). In a previous study on involuntary admission in SUD in Switzerland, we have already discussed that the requirement for coerced admissions vary greatly between different settings even within Switzerland (38). At least for our region, we could show that patient characteristics between involuntarily and voluntarily admitted patients differed regarding the behavior at admission, but not for impairment, symptoms, or functioning. The type of admission (voluntary/involuntary) does therefore not necessarily implicate presence of a more severe SUD.

Addressing clinical factors, Nordeck et al. (39) found elevated rehospitalization rates for patients with current opioid and cocaine use disorders. In our sample, however, the use of specific substances had no differential influence on readmission. This finding might be influenced by the fact that the majority of the patients of our study sample fulfilled the diagnostic criteria for more than one SUD. It can be assumed that this pattern of polyvalent substance use might have an influence on this finding: The more SUD criteria are fulfilled, the higher is the likelihood of readmission.

In our sample, more than 70% of the patients fulfilled the diagnostic criteria of another psychiatric disorder besides the SUD, defined as dual diagnosis. Interestingly, only these patients with SUD suffering from an additional psychosis or mania showed a higher risk of inpatient readmission. This finding goes along with a recent Danish study on schizophrenia and comorbid SUD confirming that SUD increases the risk of readmission (40). One reason for the higher risk of readmission might be that patients with schizophrenia and SUD show a poorer medication compliance (41), as demonstrated for the combination of bipolar disorder and SUD as well (42). But also the typical schizophrenia-associated risk factors like persistence of psychotic symptoms, lack of social support, and aggression are likely to contribute to this finding (43).

Clinical severity at discharge, as measured by the HoNOS, seems to be a valid predictor of readmission. Patients with SUD who were readmitted within the following 12 months showed a higher HoNOS score at discharge than those that were not readmitted. Tulloch et al. (44) also confirmed an association between the HoNOS score and readmission rate. A higher HoNOS at discharge might indicate a premature discharge and treatment completion is an important predictor of treatment outcome in SUD (45, 46). Furthermore, readmitted patients tend to have higher HoNOS scores at admission and a tendency to stay longer in the hospital. Nonetheless, the higher scores at discharge indicate less recovery in the group of readmitted patients.

Of all significant associations found in bivariate analysis, only the number of previous admissions and the HoNOS score at discharge remained significant in the multiple regression, while all other variables of the initial analyses were no longer statistically significant. Thus, the two factors, HoNOS score at discharge and number of previous admissions, might be considered to be the strongest predictors of readmission to SUD inpatient treatment, even after controlling for other important risk factors. In view of the fact that previous hospitalizations are consistently found to be the most important predictor of readmission, it is all the more surprising that the HONOS score at discharge further contributes to the prediction given the number of previous admissions in the model.

We believe that this finding has to be discussed in the context of the current discussions in the public mental health sector. Reimbursement of inpatient care often depends on length of stay (47), making longer inpatient treatment unattractive for institutions. Our finding provides additional evidence that this approach could increase the overall costs for the society. For healthcare providers and clinicians, this is indeed a very important topic.

Several limitations have to be taken into account when interpreting the results of our study. Readmission is an easily obtained measure of healthcare utilization and costs. Still, readmission rates serve as a proxy and should not substitute the direct assessment of outcome and program performance in patients with SUD (48). As a consequence, we also included the HoNOS in order to provide a direct assessment of outcome. Additionally, it is worth to remember that, from the clinician's perspective, an early readmission in patients with SUD might be much more helpful than a longer period without treatment in order to prevent a further worsening of the medical and social situation.

Missing data is an unavoidable problem in epidemiological research and might also affect our findings. Once the patients were discharged from inpatient treatment in the index episode, we had, for example, no information on relocation to another canton or country. We furthermore had no access to population registers which could provide information on healthcare use within the follow-up period. We therefore cannot exclude, e.g., that index patients from our study presented themselves for inpatient treatment at other hospitals. However, the PDAG is the only psychiatric institution providing acute inpatient treatment for SUDs in the canton, and in Switzerland it is obligatory that patients have to be treated within their respective canton for insurance reasons. Thus, we believe that it is rather unlikely that we missed readmissions for that reason. Still we cannot exclude that patients were, e.g., not readmitted because they suffered from severe somatic disorders or even deceased within the follow-up period.

A further limitation concerns the retrospective design of this study, which implies that our analysis was restricted to pre-registered data documented in the patient files. Our findings therefore are limited regarding the number and the kind of variables assessed. Even clinically relevant factors contributing to the outcome (directly or as confounder) therefore may be left unidentified. In the present study, we only focused on factors at the patient level. Further research on the treatment programs is required in order to analyze predictors of readmission also at an institutional level.

Regardless of these limitations, our study provides a rather long follow-up period of 1 year, which appears longer than in many other studies in the field. In addition to that, a generalization of our findings is possible because the referrals were consecutively included into our study.

Some of our findings are in contrast to previous studies. For example, we could not confirm an influence of education on readmission. Furthermore, use of opioids or cocaine was, in our sample, not associated with a significantly higher risk of readmission in comparison to alcohol. And we found no increased risk of readmission for patients with SUD and depression or other comorbidities besides psychosis. These findings underline that variables that predict readmission in one country might not necessarily apply to the next. Our main findings regarding number of admissions and HoNOS extend previous studies from other countries and underline the need to prevent patients from premature discharge in spite of a high symptom load in order to prevent readmission. Longer periods of inpatient treatment may lead to less financial compensation, making it unattractive for institutions to retain patients in an inpatient setting. But the findings of our study indicate that a higher symptom load at discharge might, on the other hand, be associated with a significantly higher risk of readmission and, accordingly, may contribute to higher costs for the healthcare providers and the society. Therefore, especially in severely affected patients and patients with more than one previous admission, it appears to be mandatory to contrast the higher risk of readmission against the incentives of a shorter length of stay. Suitable and reliable outpatient programs for severely affected patients and patients with a history of more than one inpatient treatment might contribute to resolve that dilemma.

## Data Availability Statement

Datasets will not be made available upon request due to ethical restrictions.

## Ethics Statement

The studies involving human participants were reviewed and approved by Ethikkommission Nordwest- und Zentralschweiz (EKNZ), Basel. Written informed consent for participation was not required for this study in accordance with the national legislation and the institutional requirements.

## Author Contributions

VB and BH developed this study. BL, BH, and VB performed the statistical analyzes. VB, BL, ES, WK, PR and BH discussed the findings and contributed to the manuscript.

## Conflict of Interest

The authors declare that the research was conducted in the absence of any commercial or financial relationships that could be construed as a potential conflict of interest.
